# Angiogenesis: A Pivotal Therapeutic Target in the Drug Development of Gynecologic Cancers

**DOI:** 10.3390/cancers14051122

**Published:** 2022-02-22

**Authors:** Lawrence Kasherman, Shiru (Lucy) Liu, Katherine Karakasis, Stephanie Lheureux

**Affiliations:** 1 Department of Medical Oncology, St. George Hospital, Kogarah, NSW 2217, Australia; lawrence.kasherman@health.nsw.gov.au; 2 St. George and Sutherland Clinical Schools, University of New South Wales, Sydney, NSW 2052, Australia; 3 Illawarra Cancer Care Centre, Department of Medical Oncology, Wollongong, NSW 2500, Australia; 4 British Columbia Cancer Agency, Surrey, BC V5Z 4E6, Canada; lucy.liu@bccancer.bc.ca; 5 University Health Network, Toronto, ON M5G 2M9, Canada; katherine.karakasis@uhn.ca; 6 Princess Margaret Cancer Centre, Division of Medical Oncology and Hematology, University Health Network, Toronto, ON M5G 2M9, Canada

**Keywords:** angiogenesis, tumor microenvironment, vascular endothelial growth factor, targeted therapy

## Abstract

**Simple Summary:**

Angiogenesis, defined as the abnormal development of new blood vessels in cancer, is a key component of cancer development. Clinical trials have proven that angiogenesis blockers can be effective in halting cancer growth across numerous types of gynecologic cancers. This review discusses the mechanisms of angiogenesis in gynecologic cancers, current practices and areas for development.

**Abstract:**

Since the discovery of angiogenesis and its relevance to the tumorigenesis of gynecologic malignancies, a number of therapeutic agents have been developed over the last decade, some of which have become standard treatments in combination with other therapies. Limited clinical activity has been demonstrated with anti-angiogenic monotherapies, and ongoing trials are focused on combination strategies with cytotoxic agents, immunotherapies and other targeted treatments. This article reviews the science behind angiogenesis within the context of gynecologic cancers, the evidence supporting the targeting of these pathways and future directions in clinical trials.

## 1. Introduction

It has become increasingly recognized that tumor vascularization and dysregulated angiogenesis are hallmark features of cancer development. Various angiogenic factors are implicated in the process of tumorigenesis, and within the last two decades, advances in drug development have seen the adoption of several anti-angiogenic drugs in routine oncology practice across various tumor types [1]. In gynecologic cancers, bevacizumab, a vascular endothelial growth factor (VEGF) inhibitor, has regulatory approval for use across numerous indicators within advanced-stage epithelial ovarian and cervical cancers, mostly in conjunction with chemotherapy [2]. Another angiogenesis inhibitor, lenvatinib, has also recently obtained regulatory approval, in combination with pembrozliumab, for subsequent-line treatment of advanced endometrial cancer [2]. However, despite having demonstrable benefits compared with chemotherapy alone [3,4], most patients will inevitably experience disease progression, with a proportion of patients experiencing relatively limited periods of disease control. This highlights the pressing need to develop molecular and other biomarkers to improve patient selection by predicting who may respond to these therapies, in addition to developing therapeutic strategies to overcome drug resistance.

In addition, other anti-angiogenics have demonstrated modest clinical efficacy in gynecologic cancers, although their role in standard treatment regimens remains unclear. Of these, one of the most widely studied in ovarian cancer is cediranib, an oral multi-targeted tyrosine kinase inhibitor (TKI) that has shown some clinical efficacy in recurrent ovarian cancer as a monotherapy and in combination with other drug classes [5,6,7,8]. Ongoing pre-clinical work and clinical trials will be paramount in discovering novel mechanisms for regulating tumor vascularization and improving survival in patients with gynecologic malignancies. This review discusses the known pathways and processes involved in angiogenesis, the pivotal role of dysregulation in vascularization in the context of tumorigenesis and the current therapeutic landscape in gynecologic cancers, with a focus on novel approaches to clinical trials using angiogenesis inhibitors.

## 2. Understanding Angiogenesis Pathways and the Tumor Microenvironment

### 2.1. Inducing Angiogenesis: A Hallmark of Cancer

In 2000, Hanahan and Weinberg published a framework proposing six hallmarks necessary for cancer growth and development [9]. This was updated in 2011 in line with rapid advancements in the scientific understanding of carcinogenic processes reflecting increasing diversity in targeted drug development [10]. Induction of angiogenesis has long been recognized as a cancer hallmark, with the understanding that although angiogenesis pathways exist in normal human physiology, there are various stimulatory and inhibitory mechanisms that cause dysregulation. In the existing literature, ‘angiogenesis’ refers interchangeably to all forms of neovascularization, but is classically defined as the processes of vascular sprouting, cell division, migration and the assembly of endothelial cells (EC) from pre-existing vessels [10]; throughout the remainder of this article, angiogenesis will refer to the latter definition.

Decades of pre-clinical work recognizes that angiogenesis incorporates a balance between pro-angiogenic and anti-angiogenic factors. In normal tissues, following physiological angiogenesis, its drivers usually become quiescent, and are only activated periodically in select circumstances, including wound healing or female reproductive cycling [11]. Disturbances in the balance and context of tumor growth have led to the discovery of an ‘angiogenic switch’ that is persistently activated in various cancer types [12,13]. As a result, pre-existing blood vessels are stimulated to continuously sprout new vessels to support ongoing tumorigenesis. Classically, tumor-associated vasculature are disorganized and chaotic, ignoring the standard vascular hierarchy with such abnormal features as precocious capillary sprouting, erratic blood flow, hyperpermeability and abnormal endothelial cell proliferation and behavior [14].

### 2.2. Influencing the Angiogenic Switch

Along the time spectrum of tumor growth, activation of the angiogenic switch appears to be an early event, as can be seen across several prior analyses of precursor malignant lesions such as dysplasias and in situ carcinomas [15]. Once the angiogenic switch is activated, patterns of neovascularization can differ substantially between types of cancers, ranging from mostly avascular to densely vascularized, friable tumors. After the formation of macroscopic tumors or metastasis has occurred, other peritumoral factors influence further angiogenic signaling, and tumors may even begin to adopt other modes of tumor vascularization, which are less well understood but often occur in conjunction with or independent of angiogenesis pathways [16]. Non-angiogenic vascularization mechanisms include vascular co-option, which involves tumoral hijacking of pre-existing vasculature [16,17]; intussusceptive microvascular growth, where existing vessels split to expand capillary networks [18]; and vascular mimicry, where aggressive tumor cells express stem-cell phenotypes to form de novo vascular networks [19]. The above mechanisms are less well understood than pathophysiological angiogenesis but appear to occur at differing timepoints across a tumor’s lifespan, and some are thought to occur as adaptive mechanisms to anti-angiogenic therapies [20].

Of the known pro-angiogenic factors, VEGF is the most studied in physiological and pathophysiological contexts, which explains the multitude of developmental therapeutics that target this receptor amongst others in gynecologic cancers. The VEGF family includes several gene factors which bind to VEGF tyrosine kinase receptors (VEGFR) 1-3. [21] In cancer, VEGF-A is thought to be the main stimulating factor that initiates angiogenesis through EC proliferation, migration and tube formation, upon binding to VEGFR2 on blood vessel ECs [13]. This was demonstrated in a transgenic Rip1Tag2 mouse model study of pancreatic beta-cell carcinogenesis, where the shift in growth from normal tissues to invasive carcinoma was demonstrated [13,15]. This is particularly relevant due to the well-known-to-be-hypovascularized, severely hypoxic tumoral microenvironment of pancreatic cancers, and it is thought that perhaps inhibition of angiogenesis would compromise intratumoral oxygen supply [22]. Additionally, VEGF-A inhibition suppressed tumor growth, reinforcing its key role in tumorigenesis [23].

*VEGF* gene expression is regulated by several mechanisms, of which the most important trigger is hypoxia. Oxygen levels are sensed by ECs, which primarily interact with the hypoxia-inducible transcription factor (HIF) family linked to angiogenesis, inflammation and other cellular mechanisms [24]. As hypoxia has been noted to be a feature of many cancers, consequently, elevated HIF levels are often present and portend poorer prognosis [25]. Other pro-angiogenic factors include [26,27]: fibroblast growth factors (FGF), which sustain angiogenesis through chronic upregulation; platelet-derived growth factors (PDGF); the transforming growth factor-ß (TGF-ß); bone morphogenetic proteins (BMPs); neuropilin 1 (NRP1); and hepatocyte growth factors (HGF). In contrast, examples of inhibitory factors endogenous to angiogenesis include [10,26]: thrombospondins (TSP), particularly TSP-1 which is present in the extracellular matrix; endostatin; angiostatin; angiopoietin-1 and -2; and interferon-α, -β and -γ. These inhibitors of angiogenesis are detectable in human serum under normal circumstances, suggesting that they also play a role in wound healing and preventing abnormal angiogenesis caused by emergent tumors. Additionally, non-coding microRNA (miRs), which normally play a role in regulating angiogenesis in normal tissues, are also thought to be dysregulated in some cancers. One example of this is miR-200b, which has been shown to promote metastasis and EC migration when downregulated in breast cancer [28].

Furthermore, the role of estrogen and progesterone regulation in normal endometrial physiology has been explored with regards to angiogenesis in cancer development. Numerous cell line studies have demonstrated a clear link between estrogen receptor overexpression and decreased angiogenic stimulation and, ultimately, improved prognosis [29,30], and another pre-clinical study of cervical cancer cells determined that estrogen receptor-1 loss promoted cancer invasion [31].

### 2.3. Impact of the Cellular Stroma and Tumor Microenvironment

The hypoxic environment of tumors affects peritumoral stromal cells, which in turn begets further angiogenesis and tumor growth [32]; this is particularly relevant within the pathophysiology of gynecologic malignancies [33]. Chemotactic factors secreted from cancer cells recruit immune infiltrates that secrete VEGF and other pro-angiogenic factors. These cells include tumor-associated macrophages (TAMs), neutrophils, mast cells and myeloid progenitors [34,35]. In particular, TAM functionality is highly influenced by chemokines and cytokines present in the tumor microenvironment, and can be corrupted towards an immunosuppressive, tumorigenic M2 state [35]. Other work has also highlighted the close interactions between VEGF and cancer immune evasion; for example, activation of VEGFR-1 and VEGFR-2 can suppress dendritic cell maturation and increase regulatory T cell and myeloid-derived suppressor cells, respectively [36,37]. The infiltrates of immune cells are also thought to play a role in protecting the vessels from drugs targeting EC signaling [38].

Furthermore, pericytes, which are present in normal blood vessels as supportive cells, are present sporadically in the tumor vasculature and are equally as important in this context [32]; specifically, vascular (or myeloid) progenitor cells that are recruited from the bone marrow through tumor-secreted factors have been shown to intercalate with the neovasculature as pericytes or ECs [35,39,40]. Their pathophysiological significance in this context is not completely understood, but their presence highlights the role of immune cells in upregulating angiogenesis. The immune cells that reside in the tumor microenvironment include lymphocytes, macrophages and polymorphonucleocytes [41], many of which migrate into the tumor via chemoattractants such as CSF-1, IL-3 and VEGF, and chemokines such as CCL-2 [39,42,43].

## 3. Anti-Angiogenic Agents: Clinical Data as Single Agents

Coupled with extensive pre-clinical study into the biological behavior of various gynecologic tumor subtypes, targeted therapies against VEGF activity have been developed and adopted into standard practice for advanced-stage malignancies. The following sections focus heavily on bevacizumab as it has seen the most clinical success, leading to its use as standard of care in current practice, but other angiogenesis inhibitors such as cediranib, pazopanib and ramucirumab have been trialed with varying levels of efficacy [5,44,45].

### 3.1. Bevacizumab

Bevacizumab [46] is a recombinant, humanized monoclonal antibody which targets VEGF-A. It prevents neovascularization by binding to and neutralizing the receptor, thereby inhibiting its association with endothelial receptors Flt-1 and KDR [47]. In addition, it is thought to improve the dysregulated and highly abnormal vascular structure and function associated with carcinogenesis, thereby improving the delivery of cytotoxic agents when combined with chemotherapy [48]. Despite its clear mechanism of action, no effective predictive biomarkers have been identified. For example, the three-arm, placebo-controlled, phase III GOG-218 trial enrolled 751 newly diagnosed epithelial ovarian cancer patients to receive carboplatin-paclitaxel with or without bevacizumab, and initially found no prognostic or predictive association with VEGF-A, VEGFR-2, NRP-1 or MET [49]. A subsequent blood-based biomarker analysis found that IL-6 may have had a predictive effect on bevacizumab efficacy, but the remaining pre-specified biomarkers (Ang-2, osteopontin, stromal cell-derived factor-1, VEGF-D, IL-6 receptor and GP130) did not demonstrate significant effects [50].

Bevacizumab is currently approved for use by the Food and Drug Administration [2] in combination with chemotherapy for advanced cervical, metastatic colorectal, metastatic non-small cell lung (non-squamous) and advanced epithelial ovarian cancers. In addition, bevacizumab in combination with chemotherapy has also been studied in recurrent ovarian cancer, in platinum-sensitive and resistant settings. Other uses include checkpoint inhibitor combinations for hepatocellular carcinoma, interferon-alfa combinations for metastatic renal cell carcinoma and as monotherapy in recurrent glioblastoma.

Bevacizumab monotherapy at 15 mg/kg delivered intravenously every 21 days was first evaluated in two phase 2 studies in women with recurrent epithelial ovarian cancer. In the GOG study [51], the clinical response rate was 21% and a quarter survived progression free (PFS) for more than six months. Median PFS and overall survival (OS) were 4.7 and 17 months, respectively, with reasonable tolerance. In the other study published in the same year [52], similar results were found, with overall response rates (ORR) of 15.9%, median PFS of 4.4 months and median OS of 10.7 months, although this was in a heavily pre-treated patient population who were all platinum-resistant. Overall, there was evidence of the promising activity of bevacizumab monotherapy in advanced epithelial ovarian cancer. These results led to the rationale that combination treatment with chemotherapy would be efficacious, and large phase 3 randomized studies comparing combination chemotherapy and bevacizumab to chemotherapy alone, in different settings of ovarian cancer, are discussed below.

In addition to improved survival outcomes, bevacizumab has also been demonstrated to improve the time to fluid reaccumulation, particularly with regards to symptomatic ascites. One single-arm phase 2 study enrolled 24 patients with chemotherapy-resistant ovarian cancer and administered intraperitoneal bevacizumab with each episode of paracentesis, demonstrating a 4.3-fold increase in the median paracentesis-free interval [53]. These results will be important to explore further in improving the quality of life in patients with recurrent ovarian cancer affected by ascites.

Biosimilars are compounds that are molecularly similar, but not identical, to an existing licensed and approved off-patent biologic in the market. They are intended to treat the same condition but at a lower cost. They also require a highly rigorous evaluation through clinical trials in order to establish their efficacy and safety [54]. Comparative and pharmacokineti studies must be performed before they can be approved and licensed. Of note, these are different from generics. The currently licensed and approved biosimilars of bevacizumab include ABP 215/MVASI (developed by Amgen), BCD-021 (Biocad), BI 695502 (Boehringer Ingelheim) and PF-06439535 (Pfizer) [55]. These products have been shown to be cost-effective and highly similar in efficacy when compared to bevacizumab, and have resulted in improved access to biologic therapy [56].

Bevacizumab monotherapy was also evaluated in recurrent squamous cell carcinoma of the cervix and recurrent endometrial cancer in two subsequent phase II trials [57,58]. Results from these trials indicated that whilst bevacizumab remains active in these disease sites with a reasonable tolerance, its activity as a single-agent therapy is modest and combination strategies have proven more effective.

### 3.2. Cediranib

Cediranib is an oral, potent small-molecule multi-targeted TKI targeting VEGFR-1 to -3 and c-kit. The largest monotherapy study was a phase 2 trial in North America that enrolled 74 patients with recurrent ovarian cancer, stratified into two arms according to platinum resistance [5]. The primary endpoint, ORR, was 26% in the platinum-sensitive arm, and 0% in the platinum-resistant arm, with a median PFS of 7.2 months in platinum-sensitive patients compared with 3.7 months in platinum-resistant patients. The most common grade 3/4 toxicities observed included hypertension (27%), fatigue (20%) and diarrhoea (14%). Similarly to ovarian cancer, a phase 2 cediranib monotherapy trial met its primary efficacy endpoint in endometrial cancer [59]; however, much like bevacizumab, more extensive investigations into their activity in gynecologic cancers have focused upon combination strategies. Interestingly, one phase 2 study established that cediranib use in patients with symptomatic malignant pleural effusions or ascites prolonged the time to repeated paracentesis [60].

### 3.3. Lenvatinib

Lenvatinib is another oral multi-kinase inhibitor that targets VEGFR-1 to -3, FGFR1-4, c-kit, PDGFR and the receptor that is rearranged during transfection (RET) [61]. It is approved for use in other solid malignancies as a monotherapy or a combination treatment, including thyroid, hepatocellular and renal cancers. In advanced endometrial cancer, a single-arm phase 2 study evaluated the safety and efficacy of lenvatinib in 133 patients in the subsequent-line setting, confirming a 14.3% ORR (95%CI: 8.8–21.4), with a median PFS of 5.6 months (95%CI: 3.6–7.3) [62]. The safety profile was similar to that in other cancer types, including fatigue, hypertension, nausea, anorexia and diarrhoea. Lenvatinib was assessed in five ovarian cancer patients as part of a phase 1 study of advanced solid tumors [63], however beyond this there have been no studies evaluating its activity as a monotherapy in ovarian or cervical cancers in further detail.

## 4. Combination Strategies

Due to the modest activity levels seen across numerous monotherapy studies involving anti-angiogenic agents with reasonable toxicity profiles, the bulk of clinical trials within this space have focused on combination strategies with chemotherapy, other targeted therapies or immunotherapy across all gynecologic malignancies.

### 4.1. Ovarian Cancer

**Figure 1:**
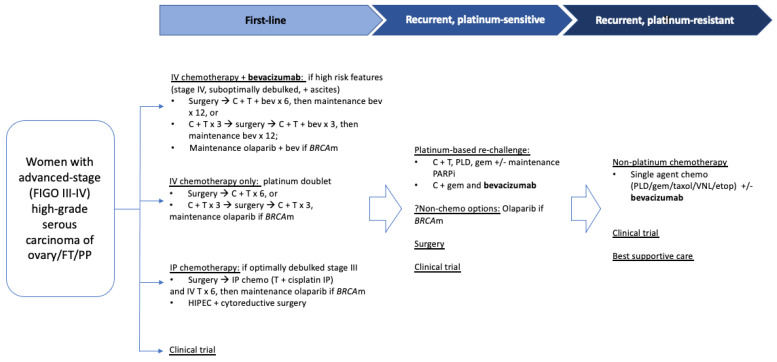
Schematic diagram of standard treatment algorithm of advanced-stage first-line and recurrent high-grade serous ovarian, fallopian tube or primary peritoneal carcinoma. Abbreviations: FT = fallopian tube; PP = primary peritoneum; C = carboplatin; T = paclitaxel; bev = bevacizumab; IV = intravenous; IP = intraperitoneal; BRCAm = BRCA mutant; HIPEC = heated intraperitoneal chemotherapy; PLD = pegylated liposomal doxorubicin; gem = gemcitabine; VNL = vinorelbine; etop = etoposide.

The importance of the tumor microenvironment in ovarian cancer—including blood and lymphoid vessels, fibroblasts, endothelial cells and immune-related cells—is being increasingly recognized for its ability to alter the surrounding stroma to facilitate tumor growth. Unfortunately, clinical activity with single-agent therapies in a recurrent disease has remained modest, leading to an increased emphasis on combining agents to target multiple pathways simultaneously. Due to the close interactions between signaling pathways within the tumor microenvironment, there is a strong rationale for further drug development and exploration of various combination therapy regimens in addition to angiogenesis inhibition, particularly in later lines, as tumors acquire various mechanisms of drug resistance (see Figure 1). Table 1 summarizes published combination phase 3 trials to date of bevacizumab in ovarian cancer.

### 4.2. Bevacizumab: First-Line

In the first-line setting, two practice-changing phase 3 randomized controlled trials were published simultaneously in the New England of Journal of Medicine in 2011: ICON7 [64] and GOG-0218 [65]. ICON7 was an international collaboration conducted across major cancer centers in Europe, Canada and Australia. This large open-label phase 3 trial was initiated after several smaller phase 2 studies had shown promising activity and demonstrated the safety of bevacizumab [51,66]. It compared the addition of bevacizumab delivered concurrently with standard chemotherapy (carboplatin and paclitaxel) intravenously every three weeks for five or six cycles, followed by an additional 12 cycles of maintenance bevacizumab or until disease progression, with standard first-line chemotherapy with carboplatin and paclitaxel alone for six cycles after cytoreductive surgery, in 1528 women with epithelial ovarian cancer across 11 countries. The primary objectives were progression-free survival, defined as the time from diagnosis to the progression/recurrence of disease or death (analyzed per-protocol), as well as interim overall survival, defined as the time from diagnosis to death of any cause. Updated PFS and OS results have since been published in 2015 [67]. The results demonstrated that, compared to standard chemotherapy alone, the addition of bevacizumab improved median progression-free survival in all patients with epithelial ovarian cancer by 2.4 months (19.5 vs. 17.5 months, *p* = 0.85), although this is no longer statistically significant in updated analysis, and there was no improvement in mean overall survival (45.5 vs. 44.6 months, *p* = 0.85). However, in an exploratory pre-planned analysis of a subgroup population with high-risk, poor prognostic features, consisting of those with stage IV disease, inoperable stage III disease or suboptimal debulked stage III disease (>1 cm residual disease after surgery), there was a statistically significant improvement in median PFS by almost 6 months (16 vs. 10.5 months, HR 0.73, 95%CI: 0.61–0.88) as well as a significant improvement in median OS by almost 10 months (39.7 vs. 30.2 months, HR 0.78, 95%CI: 0.63–0.97) [67]. Although there was more toxicity associated with the addition of bevacizumab, the authors concluded that the drug is safe when administered with chemotherapy with a manageable toxicity profile, acknowledging the higher rates of increased muco-cutaneous bleeding (37 vs. 7%), hypertension (26 vs. 7%) and thromboembolic events (11 vs. 6%). Of note, approximately 1% of enrolled patients receiving bevacizumab experienced bowel perforation, a potentially life-threatening adverse event.

Similarly, the GOG-0218 [65] study also evaluated the efficacy of bevacizumab in combination with standard chemotherapy in the first-line setting, but in a population of women with advanced stage (defined as stage III with >1 cm of residual disease post-operatively, and stage IV) epithelial ovarian cancer. This was also a large, placebo-controlled phase 3 trial conducted in several cancer centers in the United States, Canada, Japan and South Korea. Performed during the same time frame as ICON7, the study, randomized 1873 women with advanced stage ovarian cancer following debulking surgery into one of three treatment groups: a study group of bevacizumab added to chemotherapy using initiation therapy only (with a maintenance placebo), a second study group of bevacizumab added to chemotherapy used throughout the study (including maintenance bevacizumab for 15 additional treatments) and a control group of chemotherapy only followed by a maintenance placebo. The primary endpoint was also PFS. Similar to ICON7, the authors found a significant improvement in progression-free survival of 4 months (14.1 vs. 10.3 months, HR 0.717, 95%CI: 0.065–0.824) with the addition of the bevacizumab-throughout group [65]. However, updated survival results in the intention-to-treat population published in 2019 [68] did not show a statistically significant difference in OS between those treated with bevacizumab and those without it in the first line setting, with a reported median overall survival of 43.4 months in the bevacizumab-throughout group, 40.8 months in the group treated with bevacizumab concurrent with only chemotherapy and 41.1 months in the chemotherapy-with-placebo group (HR 0.96; 95%CI: 0.85–1.09; and 1.06; 95%CI: 0.94–1.20), with chemotherapy/placebo as the reference, respectively. Safety was not a major concern, although once again, a higher rate of hypertension and gastrointestinal-wall disruption was identified in the groups receiving bevacizumab.

Typically, the duration of maintenance bevacizumab after first-line treatment of advanced epithelial ovarian cancer is 15 months based on ICON7 and GOG-218. The large prospective single-arm phase 3B ROSiA study primarily evaluated the safety, but also the efficacy, of extending maintenance bevacizumab beyond 15 months in those without disease progression [67]. With over 1000 patients enrolled, 89% of them received bevacizumab beyond 15 months, with a median follow-up duration of 32 months. While the incidences of proteinuria and hypertension were higher, median PFS was reported at 25.5 months (95%CI: 23.7–27.6 months), which is the longest reported PFS in the first-line setting. However, another randomized phase 3 study presented at ASCO 2021 in the first-line setting demonstrated no benefit to PFS or OS with 30 months of bevacizumab compared to 15 months. [69] As such, standard practice remains to administer bevacizumab for 15 months in the first-line maintenance setting.

Maintenance PARP inhibitors have become a new standard of care in the past decade for women with advanced ovarian cancer after the response to platinum-based chemotherapy, although the benefit is strongest in those who have demonstrated deleterious *BRCA* mutations [70,71]. In addition, homologous recombination deficiency (HRD), where a number of other genes involved in DNA repair are dysfunctional, affects up to 50% of patients with high-grade serous ovarian cancer, and is a predictor for improved benefits from PARP inhibition [72,73]. Most recently, the combination of bevacizumab with olaparib, a PARP inhibitor, as a maintenance therapy following chemotherapy and bevacizumab has been explored in the PAOLA-1 phase 3 study for women with newly diagnosed, advanced-stage ovarian cancer [74]. Compared to bevacizumab alone, combination bevacizumab and olaparib maintenance therapy was associated with an improved PFS (HR 0.59, 95%CI: 0.49–0.72), with a median PFS of 22.1 months. This was even higher for the subgroup patient population with a somatic *BRCA* mutation and HRD, with a median PFS of 37.2 months (HR 0.33, 95%:CI 0.25–0.45), but there was no benefit seen in those with proficient homologous recombination. The concept of incorporating both therapies in the maintenance setting has become a new strategy that is increasingly attractive among patients with HRD.

### 4.3. Bevacizumab: Recurrent Setting

Bevacizumab has also been studied in patients with recurrent ovarian cancer, and the landmark phase 3 trials that led to its approval were also positive, demonstrating benefits in patients receiving combination chemotherapy and bevacizumab in this setting (without prior bevacizumab use) compared to those receiving chemotherapy only. The addition of bevacizumab to platinum-based chemotherapy in patients with platinum-sensitive recurrent ovarian cancer was associated with a significant improvement in median progression-free survival by up to four months [75,76,77]. In the platinum-resistant setting, the AURELIA study showed that combination bevacizumab with chemotherapy was associated with an improvement in PFS of three months compared to chemotherapy alone [78].

Studies evaluating bevacizumab re-treatment are scarce, and most jurisdictions allow only one line of therapy involving bevacizumab, mainly due to concerns about cost-effectiveness [79]. The phase 3 MITO16B-MaNGO study was presented at the 2018 ASCO symposium and revealed a significantly improved PFS of three months in platinum-sensitive recurrent ovarian cancer patients who were rechallenged with bevacizumab combination platinum-based doublets after previous exposure to bevacizumab in the first-line setting, without an increase in toxicity [80]. Furthermore, the aforementioned AURELIA study included 26 (7%) patients who had previously been treated with bevacizumab, and subgroup analyses confirmed similar efficacy compared with those who were bevacizumab-naïve [78]. Further confirmation of these studies is warranted. Until then, many experts in the field have argued that because bevacizumab is active in both the first-line and recurrent setting, the question is not if, but when to use bevacizumab most optimally [81]. This question becomes increasingly important when the high cost of the drug limits its widespread access.

### 4.4. Other Angiogenesis Inhibitor Combinations

Other than bevacizumab, other small molecule angiogenesis inhibitor combinations have been explored in ovarian cancer, with globally modest success. The most widely studied is cediranib, which was studied (ICON6; NCT00532194) in a randomized, double-blind, three-arm phase 3 population of 486 women with recurrent platinum-sensitive epithelial ovarian cancer [82]. Patients were randomized to chemotherapy plus placebo followed by placebo maintenance (arm A), chemotherapy plus cediranib followed by placebo maintenance (arm B) or chemotherapy plus cediranib followed by cediranib maintenance, dosed at 30 mg daily (arm C). Notably, the study was meant to proceed to a third stage powered to detect an overall survival benefit as its primary endpoint, but this was not possible due to the discontinuation of cediranib production resulting from excess toxicity in other tumor types, and the study was redesigned in September 2011 to reflect a primary endpoint of PFS, with a lower dose of 20 mg daily. There was a significant PFS benefit of arm C over arm A (HR 0.56; *p* < 0.0001); however, there were significantly worse toxicity levels in arm C, including hypertension, fatigue, diarrhoea and nausea, leading to a 20% discontinuation. An OS benefit of 7.4 months was also noted between arms C and A (27.3 vs. 19.9 months) [83]. An ongoing international phase 3 trial, ICON9 (NCT03278717), is comparing the efficacy of 300 mg of maintenance olaparib twice daily versus 20 mg of olaparib plus cediranib daily. Another phase 3 study compared the combination of 30 mg of cediranib daily and olaparib as a treatment in relapsed, platinum-sensitive high-grade serous or endometrioid ovarian cancer to platinum-based chemotherapy, and did not meet its primary PFS endpoint, with similar concerns about toxicity [7]. Results in the platinum-resistant context are pending (GY005; NCT02502266).

In the recurrent ovarian cancer setting, pazopanib, a multitargeted TKI affecting VEGFR, PDGFR and c-Kit, was evaluated in a randomized phase 2 trial in 74 patients with a platinum-resistant disease in combination with paclitaxel, demonstrating a 3-month improvement in PFS (HR 0.42; *p* = 0.002) [84]. Trebananib, a peptibody that blocks angiopoietin-1 and -2 binding to Tie2, showed PFS but no OS benefit combined with paclitaxel compared to paclitaxel alone in a phase 3 trial of 461 patients [85,86]. Similarly, these findings were also reflected in the first-line ovarian cancer population in the phase 3 TRINOVA-3 trial when combined with platinum-doublet chemotherapy [87].

#### 4.4.1. Cervical Cancer

Angiogenesis is well known to be a key component of carcinogenesis in cervical cancer and its precursor lesions [88]. Unique to cervical cancer is its strong causative association with human papillomavirus. Specifically, the production of the E6 protein contributes to the dysregulation of p53 induction and ubiquitination, leading to p53 protein degradation and, consequently, VEGF upregulation [89]. Furthermore, the E7 protein displaces histone deacetylases HDAC1, HDAC4 and HDAC7, upregulating HIF1α and consequently increasing VEGF production [90].

Similar to ovarian cancer, bevacizumab is the most widely studied anti-angiogenic agent, and the only one which has seen clinical success in the advanced setting. GOG 240 was a phase 3 randomized trial that enrolled 452 women with recurrent or metastatic cervical carcinoma, previously untreated, to receive either a platinum-doublet (cisplatin-paclitaxel) or non-platinum-doublet (topotecan-paclitaxel), with or without bevacizumab [91]. In the platinum arms, the study met its primary endpoint, demonstrating a median OS benefit of 3.7 months (17.0 vs. 13.3 months; HR 0.71; *p* = 0.004) and higher ORR (48 vs. 36%, *p* = 0.008) with the addition of bevacizumab. There were no significant differences in quality-of-life outcomes between treatment arms [92]. The results of this trial, in addition to a systematic review of 23 studies comparing bevacizumab and non-bevacizumab containing regimens, [93] have led to the regulatory approval and routine implementation of bevacizumab with platinum-doublet chemotherapy in the first-line setting. In terms of safety, although most adverse events had similar rates for ovarian cancer patients, risk of gastrointestinal and genitourinary fistulae was noted to be significantly higher (13 vs. 1%; odds ratio 17.50) [91], and subsequent cohort studies reinforce that caution should be taken, particularly in those with recurrent pelvic disease who previously received pelvic radiotherapy [94,95].

Beyond bevacizumab, published data assessing angiogenesis inhibitors in advanced cervical cancer is limited to phase 1 or 2 studies. The CIRCCa trial was a randomized, double-blind, placebo-controlled phase 2 trial of patients with a recurrent or metastatic disease who received carboplatin and paclitaxel, followed by either 20 mg of cediranib daily or the placebo until disease progression [96]. Of 69 patients enrolled, the primary endpoint PFS was significantly longer in the cediranib group (8.1 vs. 6.7 months, HR 0.58; *p* = 0.032), but with a significant increase in toxicity. Other small molecule TKI studies have looked at the activity of pazopanib, lapatinib, sunitinib and imatinib, all with somewhat disappointing results [97,98,99].

#### 4.4.2. Endometrial Cancer

Across the molecular spectrum of endometrial cancer, elevated levels of pro-angiogenic factors such as VEGF, platelet-derived EC growth factor (PD-ECGF) and FGF are prognostic for survival [100]. Additionally, other factors, including capillary network density, HIF1α expression and tumor hypoxia, are also known to be associated with endometrial cancer growth, although clinical trial results in this space have shown mixed results [101]. Although the addition of bevacizumab to chemotherapy showed disappointing results in multiple phase 2 studies and thus has not been evaluated in a phase 3 setting, lenvatinib in combination with pembrolizumab, a programmed cell death-1 (PD-1) inhibitor, has demonstrated clinical success [4]. Clinical efficacy of this TKI/PD-1 inhibitor combination in ovarian cancer is also currently being explored, with results eagerly awaited.

Bevacizumab combined with carboplatin and paclitaxel was assessed in a randomized phase 2 study of 108 patients with treatment-naïve, advanced-stage or recurrent endometrial cancer [101]. Patients were given 6–8 cycles of chemotherapy and bevacizumab or placebo, followed by maintenance until disease progression. The study did not meet its primary endpoint, with a trend towards, but no statistically significant increase in, PFS (HR 0.84; *p* = 0.43) and OS (HR 0.71; *p* = 0.24). Other combination phase 2 studies with chemotherapy such as pemetrexed have also shown only modest activity [102].

The combination of lenvatinib and pembrolizumab is currently being trialed across a variety of tumor types, and initially was assessed in a phase 1b/2 study, KEYNOTE-146, within a cohort of 108 pre-treated, advanced-stage endometrial cancer patients [103]. The primary endpoint of a 24-week ORR was met (38%, 95%CI: 28.8 to 47.8%) and activity was demonstrated regardless of mismatch repair (MMR) status, which is a known marker predictive of response to immunotherapy. A follow-up phase 3 study, KEYNOTE-775, which enrolled 827 women who had progressed after one platinum-based regimen to receive either lenvatinib and pembrolizumab or a physician’s choice of chemotherapy, met its co-primary endpoints of PFS and OS [4,8]. Median PFS was 7.2 vs. 3.8 months (HR 0.56; *p* < 0.0001) and median OS was 18.3 vs. 11.4 months (HR = 0.63; *p* < 0.0001). 697 (84.3%) patients were MMR-intact, and the differences between arms were preserved in this population subgroup of interest (PFS HR 0.60, *p* < 0.0001; OS HR 0.68, *p* < 0.0001) [4]. Adverse events in the experimental arm reflected those from previous studies, with 88.9% of patients experiencing grade 3 or higher adverse events, with the most common being hypertension (64%), hypothyroidism, diarrhea, nausea, anorexia, vomiting, decreased body weight and fatigue. Notably, arthralgia of any grade was noted in 30.5% of patients in the experimental arm compared with 8% in the chemotherapy arm. Furthermore, the supplementary appendix highlighted a 1% incidence of abdominal pain recorded as a serious adverse event in the experimental arm, compared with 0.3% in the chemotherapy arm. These findings have been practice-changing, earning regulatory approval of this combination in subsequent-line therapies for advanced endometrial cancers [2], and highlight that targeting synergistic pathways in the tumor microenvironment is a clinically viable route for drug development in these patients.

## 5. Angiogenesis Inhibitors in Gynecologic Cancers: Carving a New Path

**Figure 2:**
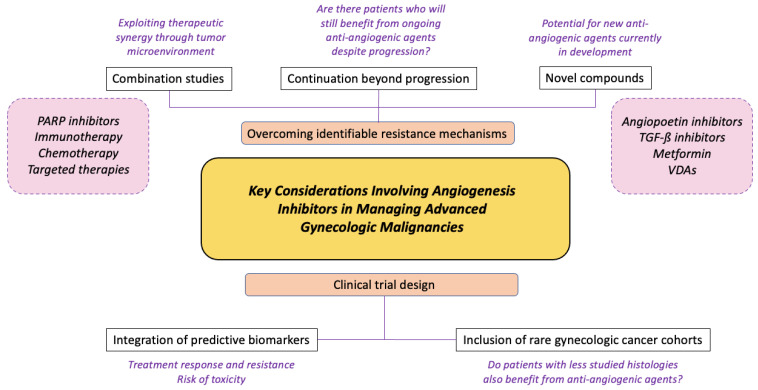
Schema of key considerations involving angiogenesis inhibitors in managing advanced gynecologic malignancies. Angiogenesis inhibitors are currently integral to the management of advanced gynecologic cancers; however, there is a definite therapeutic ceiling effect, particularly in later lines of treatment. Key considerations include identification and evasion of potential resistance mechanisms, coupled with integration of particular concepts into clinical trial design. Abbreviations: PARP = poly-ADP ribose polymerase; TGF-ß = transforming growth factor-beta; VDAs = vascular disrupting agents.

The scientific community has seen significant advances over the last decades in their understanding of tumor biology and, consequently, drug development, with angiogenesis agents being incorporated into clinical trials across the spectrum of gynecologic malignancies. Despite these victories, disease-specific mortality in advanced-stage disease remains unacceptably high. At present there appears to be a ceiling effect, where the benefits of continuing anti-angiogenic agents across multiple treatment lines are questionable. New data are coming to light showcasing the ongoing activity of bevacizumab beyond disease progression in ovarian cancer [80]. While there is no doubt that angiogenesis inhibitors are likely to continue to remain crucial to therapeutic development in future studies, several clinical concepts need to be considered (Figure 2).

One therapeutic strategy that has proven effective is examining the effects of different drug class combinations to produce better clinical outcomes. While pre-clinical work has elucidated the clear role of abnormal angiogenesis in gynecologic cancer growth and development, clinical efficacy of VEGF inhibitors such as bevacizumab is not universal, and even those patients who respond initially can rapidly develop resistant clones [104]. The current knowledge of definite resistance mechanisms include the modulation of other non-VEGF-related angiogenesis mechanisms, immunogenic pathways, tumor hypoxia, VEGF overexpression and vascular pericyte overpopulation [104,105]. In addition, in ovarian cancer, there appears to be certain molecular subgroups [106] of patients that are more likely to benefit from bevacizumab [107]. Currently, there are no biomarkers validated to select patient who will benefit from bevacizumab, and further work is needed.

Similarly, the scientific understanding of biomarkers predictive of responses is constantly evolving. In endometrial cancers, it is likely in the future that biomarkers will guide further subclassifications by therapeutic agent; recent publications have explored differential responses between patients across the spectrum of MMR deficiencies with pembrolizumab [108,109]. For those who are less likely to have deep, prolonged responses to immunotherapy, KEYNOTE-775 [8] suggests that lenvatinib may be more useful in this situation, but this needs to be explored further. Careful dissection of biomarkers in endometrial cancer, such as those differentiating between somatic MMR deficiency and germline MLH1 methylation [108], may provide interesting results in future trials.

**Figure 3:**
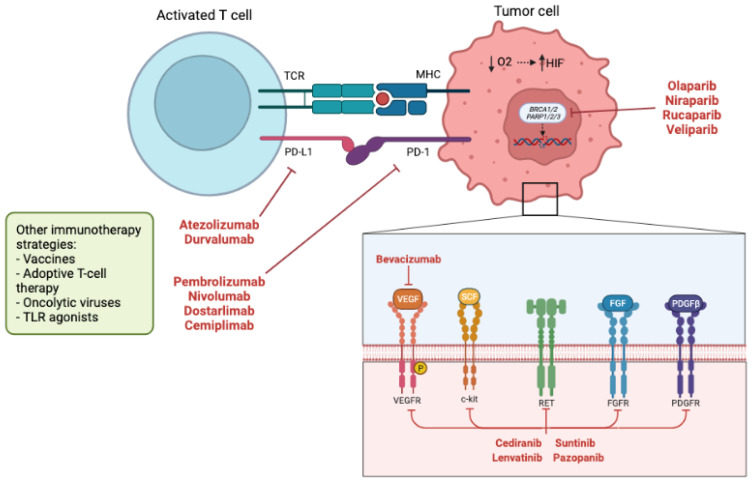
Schematic diagram of current therapeutic targets in gynecologic cancers. Efforts are currently focusing on the tumor microenvironment with regards to immune modulation and angiogenesis inhibition pathways. Created with Biorender.com. Abbreviations: TCR = T cell receptor; MHC = major histocompatibility complex; PD-L1 = programmed cell death ligand-1; PD-1 = programmed cell death-1; HIF = hypoxia inducible factor; BRCA = breast cancer gene; PARP = poly ADP ribose polymerase; TLR = toll-like receptor; VEGF = vascular endothelial growth factor; RET = rearranged during transfection receptor; FGF = fibroblast growth factor; PDGF = platelet derived growth factor.

Clinical trials are looking to overcome proposed resistance mechanisms by regulating other components of the tumor microenvironment, such as the immune system and cell cycle checkpoints (Figure 3). In epithelial ovarian cancer, studies of the front-line and recurrent settings have demonstrated promising synergy using PARP inhibitors [7,74,110] with immunotherapy [111,112]; consequently, numerous phase 3 trials are underway in the front-line setting to examine combination chemotherapy, bevacizumab, immune checkpoint inhibitors and PARP inhibitors (NCT03737643, NCT03740165, NCT03522246, NCT03602859), most of which should achieve primary study completion and have preliminary results available within the coming five years.

Similarly, in a recently published phase 3 trial of 548 patients with advanced or recurrent cervical carcinoma, the addition of pembrolizumab to chemotherapy with or without bevacizumab demonstrated statistically significant benefits for co-primary endpoints, with a median PFS of 10.4 vs. 8.4 months (HR 0.62; *p* < 0.0001), and an OS at 24 months of 50.4 vs. 40.4% (median not reached; HR 0.67; *p* < 0.001) [113]. More than 60% of patients in both arms used bevacizumab.

However, not all large-scale combination studies have shown favorable results. IMagyn050 was a phase 3 study of 1301 patients with newly diagnosed epithelial ovarian cancer who received carboplatin, paclitaxel, bevacizumab with either atezolizumab, a PD-L1 inhibitor or a placebo [114]. The study did not meet its primary endpoint of PFS in the intention-to-treat or PD-L1-positive populations, and immature OS results were also not statistically significantly different. This contrasts with prior early-phase studies with immune checkpoint inhibitors plus bevacizumab that showed promise [111,115], and thus subsequent phase 3 studies (NCT03353831, NCT02891824) in the recurrent setting will further delineate the role that this combination has in treating ovarian cancer. These disparate results highlight the importance of careful patient selection in trial design, which will become more apparent through ongoing predictive biomarker discovery.

Many of the trials discussed in this review are promising and are likely to alter the clinical therapeutic landscape of gynecologic cancers in the years to come. However, caution with regards to cumulative toxicity, careful patient selection, education and monitoring of knowledge translation for clinical practice needs to be considered. One example of the importance of patient monitoring is the previously discussed KEYNOTE-775 study in advanced-stage endometrial cancer, where lenvatinib and pembrolizumab led to a grade 3 treatment-emergent adverse event (TEAE) rate of 89%, with 14% of patients discontinuing both drugs due to a TEAE [4,8]. Due to the substantial rates of adverse events, oncologist-led directives are trialing lower doses of lenvatinib and finding similar efficacy outcomes, with improved toxicity [116]. Similarly, in the ICON6 study, the doses of cediranib in the combination arms had to be reduced due to toxicity, and fortunately this was found to be more tolerable for patients [82]. Thus, particularly in the real-world setting where patient performance status or comorbidities may be suboptimal, patients will require thorough counselling of toxicity risks prior to commencing novel combination treatments, and it is likely that further studies in real-world settings will be required to assist clinicians about optimal dosing regimens and alterations.

The incorporation of flexible trial designs with exploratory cohorts for rare gynecologic cancers, and the integration of biomarkers into trial conduct, are necessary to select patients who are likely to benefit from treatment. In ovarian and cervical cancers, whilst most suitable patients seem to benefit from the addition of bevacizumab to front-line chemotherapy when indicated [67,68], there are those who only have modest responses to combination therapy, and at present there are no effective biomarkers to differentiate who is more likely to respond well to bevacizumab. In endometrial cancer, although the addition of lenvatinib to pembrolizumab created therapeutic synergy to produce favorable outcomes even in MMR-proficient patients, the duration of response ranged from 1.6 to 23.7 months, with a median of 9.2 months [4].

Thus, is there a biomarker that can predict who is likely to have a more durable response? In the context of PARP inhibitors and ovarian cancer, it is well established that *BRCA* mutations and HRD positivity predict for more sustained progression-free periods [117]; ideally, in the future, the development of such predictive markers for response, resistance and toxicity will be available for anti-angiogenic agents, as well for the integration of real-time molecular sequencing and tumor profiling into care. Furthermore, with the inclusion of exploratory cohorts and adaptive study designs of rarer, less well-studied histologies, such as non-epithelial ovarian cancers [118,119], mucinous gynecologic tumors and carcinosarcomas, this could potentially provide further insights into disease biology and therapeutic activity in conditions that would otherwise be difficult to study in standalone trials. Even though patient numbers from phase 2 trials of recurrent sex-cord stromal tumors are small due to their rarity, the administration of bevacizumab in 36 patients demonstrated an ORR of 16.7%, with 77.8% having stable disease, and a median PFS of 9.3 months; these results confirm the activity of bevacizumab in this patient cohort [119].

While anti-VEGF, PDGF and EGFR therapies have proven somewhat effective in specific patient populations, many other angiogenesis pathways remain relatively unexplored in the context of drug development. Agents targeting the angiopoietin pathway, such as trebananib, and the TGF-ß pathway, such as tasisulam, have been met with limited clinical success [85,86,87,120]. The normalization of tumor vascularization through the upregulating of thrombospondin-1and the promotion of smooth muscle cell proliferation and migration in conjunction with EC apoptosis could potentially regulate therapeutic intratumoral drug delivery, enhancing efficacy [121,122]. Drug development in this area appears promising, but remains in pre-clinical stages at present [123].

Vascular disrupting agents (VDAs) are a novel class of anti-angiogenic agents that target EC architecture and can be split into two types: combretastatin A4-phosphates (CA4P) and flavonoids [124]. The only VDA in development for ovarian cancer is fosbretabulin, a CA4P prodrug which has been assessed in two phase 2 studies in combination with bevacizumab or pazopanib, with modest but promising results [125,126]. In cervical cancer, vadimezan (DMXAA) is the only flavonoid or VDA that has been tested in a clinical context [127]. Other agents are in development that have yet to be tested in clinical trials [128,129,130]; currently, it is too early to tell whether these will have a meaningful impact upon the therapeutic landscape.

## 6. Conclusions

Over the last decade, the adoption of angiogenesis inhibitors in the standard care of gynecologic cancers represents decades of monumental advances in pre-clinical, translational and clinical medicine. As a hallmark of cancer development, though the concept of abnormal angiogenesis is well understood, there are many aspects of the angiogenesis pathway that remain untapped in the context of drug development. While bevacizumab and its biosimilars are now entrenched in systemic therapy regimens in ovarian and cervical cancers, there remains plenty of room for exploration within these areas, particularly as biomarkers are developed to inform patient selection for therapy. Significant advances have been demonstrated targeting angiogenesis in synergy with immune checkpoint inhibition in endometrial cancer, and ongoing combination trials using novel anticancer drugs, combination studies with adaptive trial designs and integrated biomarker studies remain key cornerstones in further advancing this area of oncological research, keeping the ultimate goal of improving survival outcomes, mitigating toxicity and optimizing patient quality of life with these therapies.

## Figures and Tables

**Table 1:** Summary of presented combination phase 3 trials with bevacizumab in ovarian cancer.

Trial	Arms	Sample Size	Patient Characteristics	PFS			OS		
				Median (mo)	HR	95%CI	Median (mo)	HR	95%CI
ICON7	CT	764	Newly diagnosed	17.5	0.93	0.83–1.05	58.6	0.99	0.85–1.14
	CT + Bev + mBev	764		19.9			58.0		
ICON7	CT	254	Newly	10.5	0.73	0.61–0.88	30.2	0.78	0.63–0.97
	CT + Bev + mBev	248	diagnosed High risk	16.0			39.7		
GOG-0218	CT + P + mP	625	Newly diagnosed Stage III-IV	10.3	0.717; 0.908	0.625–0.824; 0.795–1.040	41.1	0.96; 1.06	0.85–1.09; 0.94–1.20
	CT + Bev + mBev; CT + Bev + mP	623; 625		14.1; NR			43.4; 40.8		
GOG-0262	CT + Bev + mBev	289	Newly diagnosed Stage III-IV	14.7	0.99	0.83–1.20	40.2	0.94 (all)	0.72–1.23
	ddCT + Bev + mBev	291		14.9			39.0		
GOG-0262	CT + Bev + mBev	298	Newly diagnosed Stage III-IV	14.7	0.70	0.625–1.173	NA	NA	NA
	CT	57		10.3			NA	NA	NA
GOG-0262	ddCT + Bev mBev	291	Newly diagnosed Stage III-IV	14.9	0.95	0.690–1.385	NA	NA	NA
	ddCT	55		14.2			NA		
PAOLA-1	CT + Bev + mBev + mP	267	Newly diagnosed Stage III-IV	16.6	0.59	0.49–0.72	NA	NA	NA
	CT + Bev + mBev + mOlaparib	537		22.1			NA		
PAOLA-1	CT + Bev + mBev + mP	80	Newly diagnosed stage III-IV, sBRCA+	21.7	0.31	0.20–0.47	NA	NA	NA
	CT + Bev + mBev + mOlaparib	161		37.2			NA		
OCEANS	CG + P + mP	242	Platinum-sensitive ROC	8.4	0.484	0.388–0.605	32.9	0.95	0.77–1.18
	CG + Bev + mBev	242		12.4			33.6		
GOG-0213	CT	337	Platinum-sensitive ROC	10.4	0.628	0.534–0.739	37.3	0.829	0.683–1.005
	CT + Bev + mBev	337		13.8			42.2		
ENGOT OV.18	CG + Bev + mBev	337	Platinum-sensitive ROC, prior Bev (41%)	11.7	0.807	0.681–0.956	28.2	0.833	0.680–1.022
	CD + Bev + mBev	345		13.3			33.5		
MITO16b	CT/CG/CD	203	Platinum-sensitive ROC, prior Bev (100%)	8.8	0.51	0.41–0.65	27.1	0.97	0.70–1.35
	CT/CG/CD + Bev	202		11.8			26.7		
AURELIA	wT/D/topotecan	182	Platinum-resistant ROC, <3 prior line	3.4	0.42	0.32–0.53	13.3	0.85	0.66–1.08
	wT/D/topotecan + Bev	179		6.7			16.6		

Abbreviations: PFS = progression-free survival; OS = overall survival; mo = months; HR = hazard ratio; 95%CI = 95% confidence interval; CT = carboplatin and paclitaxel; Bev = bevacizumab; mBev = maintenance bevacizumab; P = placebo; mP = maintenance placebo; ddCT = dose dense carboplatin and paclitaxel; mOlaparib = maintenance olaparib; CD = carboplatin and liposomal doxorubicin; CG = carboplatin and gemcitabine; wT = weekly paclitaxel; D = liposomal doxorubicin; ROC = recurrent ovarian cancer.
